# One-Year Lesson: Machine Learning Prediction of COVID-19 Positive Cases with Meteorological Data and Mobility Estimate in Japan

**DOI:** 10.3390/ijerph18115736

**Published:** 2021-05-27

**Authors:** Essam A. Rashed, Akimasa Hirata

**Affiliations:** 1 Department of Electrical and Mechanical Engineering, Nagoya Institute of Technology, Nagoya 466-8555, Japan; ahirata@nitech.ac.jp; 2 Department of Mathematics, Faculty of Science, Suez Canal University, Ismailia 41522, Egypt; 3 Center of Biomedical Physics and Information Technology, Nagoya Institute of Technology, Nagoya 466-8555, Japan

**Keywords:** COVID-19, forecasting, LSTM, meteorological data, deep learning

## Abstract

With the wide spread of COVID-19 and the corresponding negative impact on different life aspects, it becomes important to understand ways to deal with the pandemic as a part of daily routine. After a year of the COVID-19 pandemic, it has become obvious that different factors, including meteorological factors, influence the speed at which the disease is spread and the potential fatalities. However, the impact of each factor on the speed at which COVID-19 is spreading remains controversial. Accurate forecasting of potential positive cases may lead to better management of healthcare resources and provide guidelines for government policies in terms of the action required within an effective timeframe. Recently, Google Cloud has provided online COVID-19 forecasting data for the United States and Japan, which would help in predicting future situations on a state/prefecture scale and are updated on a day-by-day basis. In this study, we propose a deep learning architecture to predict the spread of COVID-19 considering various factors, such as meteorological data and public mobility estimates, and applied it to data collected in Japan to demonstrate its effectiveness. The proposed model was constructed using a neural network architecture based on a long short-term memory (LSTM) network. The model consists of multi-path LSTM layers that are trained using time-series meteorological data and public mobility data obtained from open-source data. The model was tested using different time frames, and the results were compared to Google Cloud forecasts. Public mobility is a dominant factor in estimating new positive cases, whereas meteorological data improve their accuracy. The average relative error of the proposed model ranged from 16.1% to 22.6% in major regions, which is a significant improvement compared with Google Cloud forecasting. This model can be used to provide public awareness regarding the morbidity risk of the COVID-19 pandemic in a feasible manner.

## 1. Introduction

Since its outbreak from Wuhan City, China, coronavirus disease (COVID-19) has spread into a global pandemic. COVID-19 has caused severe global damage to humanity with 140 million recorded infections and over 3 million deaths (https://coronavirus.jhu.edu (accessed on 19 April 2021)). A novel virus with a high infection rate has a strong effect on daily life activities as well as the economic cycle. Therefore, future forecasting of an outbreak would enable a balance between public protection and economic revival. Several studies have discussed different factors that correlate with the spread of COVID-19. It is clear that public mobility and meteorological factors are highly correlated with the pandemic in many regions worldwide [1,2,3,4,5]. Based on a recent study on data from China, high-density cities (with potentially more mobility factors) were found to be at a higher risk of SARS-CoV-2 infection [6]. However, how these factors determine the spread of the pandemic remains controversial and under discussion.

Different models have been designed to predict future positive/death cases [7,8,9,10,11,12]. Machine learning and other artificial intelligence techniques are expected to provide better data analysis and lead to its understanding as well as provide more accurate prediction models [11,13,14,15,16,17,18,19,20]. Infectious diseases are commonly analyzed using compartmental models that define the pipeline of the whole cycle, including infection, treatment, recovery, and death. Susceptible-exposed-infectious-recovered (SEIR) is a conventional model that was used to represent COVID-19 [21]. The SEIR model was adopted as detailed in [22] to estimate COVID-19 cases in the United States and became available for public use (http://g.co/covidforecast (accessed on 12 January 2021)). In mid-November 2020, Google announced an online dashboard that provides a 28-day forecasting of COVID-19 spread in Japan. The dashboard provides predictions of the number of confirmed positive cases, deaths, hospitalized patients, and recovered patients. While the prediction data may provide interesting highlights for the management of healthcare resources, government policies, and actions required, a large variation is observed in the prediction with frequent updates. The Google model is based on a machine learning framework that considers mobility reports as a reference for potential social contact that is known to influence the spread of the infection. In our earlier studies, we confirmed that meteorological factors such as temperature and humidity are also correlated with the spread/decay of pandemics in Japan [23,24], as well as other countries [25].

In this study, we propose an alternative model that considers different factors and study the efficiency of predicting COVID-19 cases. A deep learning model employing a long short-term memory (LSTM) neural network [26] was designed for predicting positive COVID-19 cases in the future. LSTM is known to be a very efficient model for time-series data analysis and forecasting compared to conventional regression-based models. We validated the proposed model using data acquired from regions with high infection records in Japan.

## 2. Related Work

With the availability of COVID-19 data, several groups have analyzed different factors and formulated a variety of forecasting models. An early work forecasted positive cases in India [27] using data from January to April 2020. The error percentage computed using the total number of positive cases was less than 20%. In addition, several LSTM neural networks were compared to estimate the number of daily/weekly positive cases (DPC/WPC) in India from March to May 2020 [28], suggesting that bi-directional LSTM demonstrated the best performance. The absolute percentage error of the total positive cases ranged from 3–5% for different LSTM architectures.

A comparative study using data from India and the USA indicated that convolutional LSTM models outperform stacked convolutional LSTM models when data from February to July 2020 were used [29]. An architecture consisting of an LSTM network and a fully connected (FC) network was used for the prediction of COVID-19 incidences in Iran using data from Google Trends (GT) [30]. Daily new positive cases between February and March 2020 were used to validate the proposed network. The root mean square error computed from a 10-fold cross-validation study was reported to be 27.187. A study conducted using the cumulative number of confirmed cases in Isfahan, Iran, between January and May 2020 was used to test different machine learning forecasting models [31]. The input data included DPC and social determinants of health (SDH). The mean absolute percentage error of the LSTM model was reported to be 2.41%.

Data from Russia, Peru, and Iran obtained from January to July 2020 were used to validate a standard LSTM network [32]. The daily mean square error of the cumulative positive cases was less than 6%. Another study considered data from Canada to provide forecasts of different sets of future days and predicted the end of the COVID-19 outbreak to be around June 2020 [33].

Overall, a straightforward comparison is not feasible because different studies use different metrics to compute the error, and errors were computed for cumulative data (not daily new cases) in some papers. Table 1 summarizes the different characteristics of these studies.

While the above studies provide frameworks for the use of LSTM networks in COVID-19 forecasting, they are still limited from different perspectives. First, all models were trained using earlier data of positive cases without considering other factors, such as meteorological factors and public mobility. This can be sufficient when the training/testing data are limited with no significant change in weather data or public activities. However, it may not be efficient with long-term forecasting tasks with different restrictions. Second, the lack of ablation studies on different LSTM structures limits the prediction model to a small set of standard structures. Therefore, it may be useful to provide a more general framework that considers related factors and study a new LSTM network design with a long-term data record.

## 3. Materials and Methods

### 3.1. Daily Positive Cases

The number of DPCs of COVID-19 in Japan were obtained from online open data sources provided by the Japanese Ministry of Health, Labour, and Welfare (https://www.mhlw.go.jp/stf/covid-19/open-data.html (accessed on 12 January 2021)) and websites of local prefectures.

### 3.2. Meteorological Data

**Figure 1:**
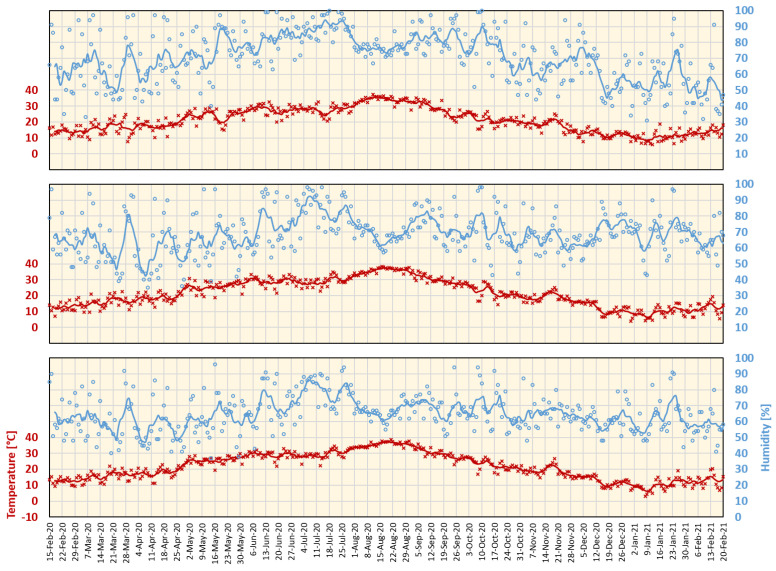
Daily maximum temperature and average humidity for Tokyo (**top**), Aichi (Nagoya) (**middle**), and Osaka (**bottom**) from 15 February 2020 to 20 February 2021. Lines represent a 7-day average.

The meteorological data covering the study period were extracted from the Japan Meteorological Agency (JMA) online resource (https://www.jma.go.jp/jma/index.html (accessed on 1 March 2021)). Meteorological data for Tokyo, Aichi, and Osaka are shown in Figure 1.

### 3.3. Mobility Data

**Figure 2:**
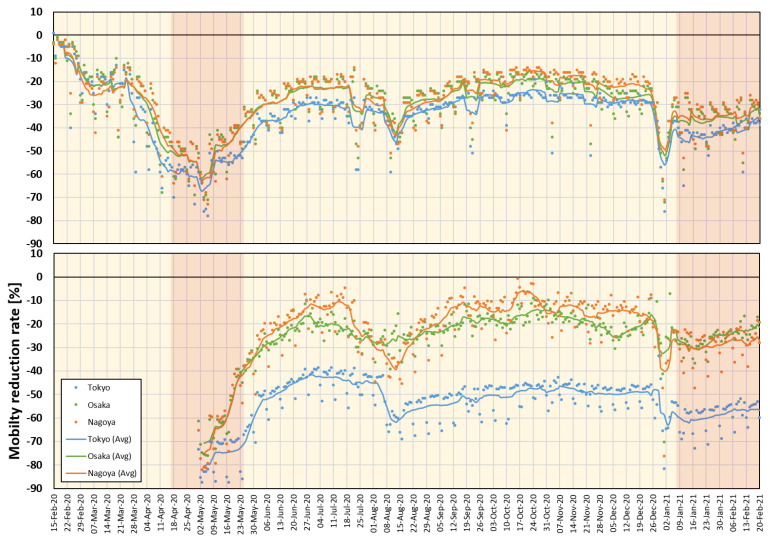
Mobility reduction rate according to the Google mobility report (**top**) and DoCoMo mobile usage (**bottom**) in Tokyo, Aichi (Nagoya main station), and Osaka. Red regions demonstrate the time for Japan’s national state of emergency. Lines represent a 7-day average.

The proposed model considers public mobility information, which is mainly estimated using mobile use data around major spots all over Japan. These data are available for 95 different local points and are updated daily by major mobile phone carriers (NTT DoCoMo, Inc. (Tokyo, Japan)) that have approximately 37.3% market share. These data represent the percentage of reduction in network connections of mobile devices compared to the same day before the pandemic. Major train stations may provide a good estimation of public mobility and can be used as a surrogate of social distancing [23], which has been confirmed to be a major factor contributing to the worldwide spread of the pandemic. Mobility data from 1 May 2020, were obtained from the NTT DoCoMo mobile use statistics (https://mobaku.jp/covid-19/ (accessed on 1 March 2021)), and earlier data (15 February 2020 to 30 April 2020) were obtained from Google mobility reports (https://www.google.com/covid19/mobility/ (accessed on 1 March 2021)). In Google mobility reports, the “transit_stations_percent_change_from_baseline” is considered as a surrogate value that is equivalent to the NTT DoCoMo mobility data. The mobility data for, Aichi, and Osaka are shown in Figure 2.

Three geographic regions that represent major urban areas with high population and major infection rates in Japan were selected (Table 2). Additional data from three regions (Hyogo, Kyoto, and Fukuoka) were also used to confirm the applicability of the proposed framework over a wider range. It is worth noting that many other climate factors such as pollution level, UV, wind, and precipitation have been discussed in other studies (e.g., [34,35]) as potential correlated factors. However, we have found that the influence of these factors is marginal, at least in Japan [23,24], which is consistent with [36] and is thus excluded here.

### 3.4. Google Cloud Forecast

**Figure 3:**
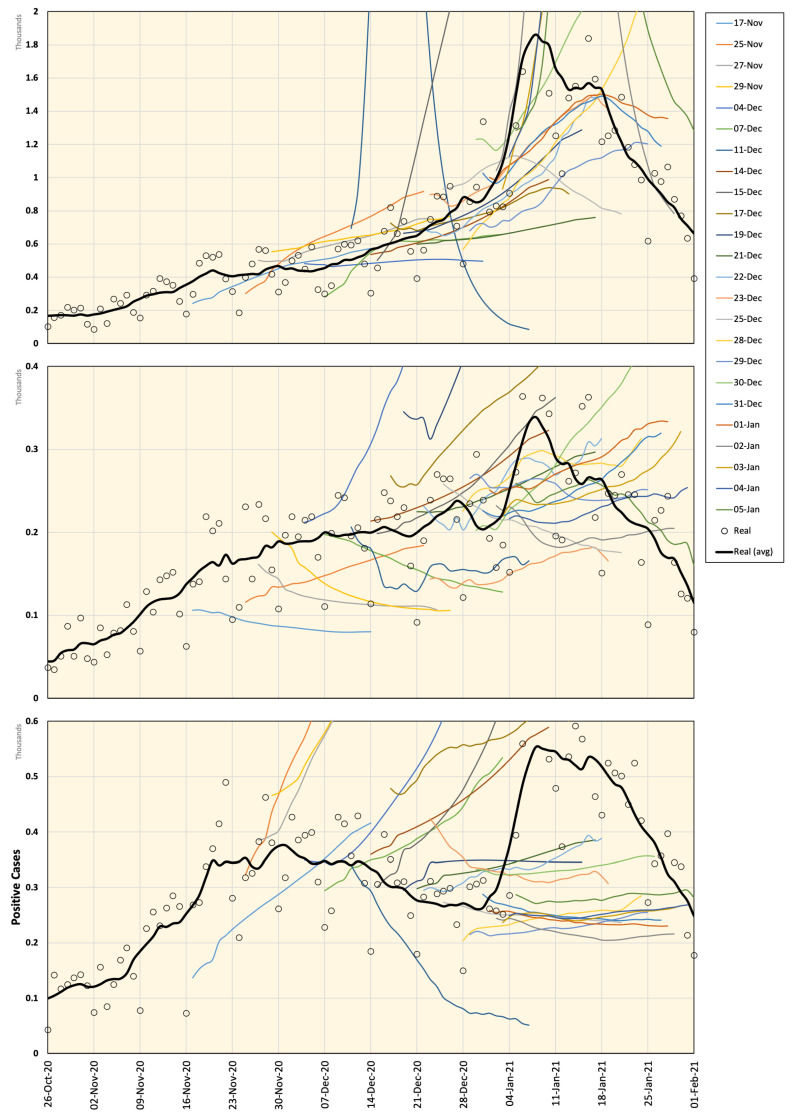
Google Cloud forecasting of DPC in Tokyo (**top**), Aichi (**middle**), and Osaka (**bottom**). Different versions are labeled with the forecasting start date and compared to the real values recorded from 26 October 2020 to 25 January 2021. All curves represent a 7-day average value.

Google Cloud forecast data for Japan are available online (https://datastudio.google.com/s/jbtyZdv8uwI (accessed on 7 January 2021)) and are updated regularly (non-periodic updates). The Goggle model is based on the integration of machine learning into compartmental disease models. Specifically, the standard SEIR model [37] was adopted to handle forecasting COVID-19 data. The model demonstrates potential case transitions in different phases, such as susceptible, infected, hospitalized, recovered and dead. The model is demonstrated by several equations and parameters estimated from the training data. Details can be found in [22]. As the history of prediction updates is no longer available online, we decided to keep an offline record of the prediction data samples to study the consistency and reliability of the prediction compared to actual recorded cases when available. Samples of the prediction history of positive COVID-19 cases in Tokyo, Aichi, and Osaka are plotted in Figure 3. It is clear that, in many instances, an excessive number of positive cases are estimated with significant error values. Moreover, the inconsistency of the data is relatively high. For example, the estimated data pattern is inverted (increasing/decreasing) within a short time period (a day or two). These flipping patterns may confuse decision makers regarding the expected scenarios and required actions.

### 3.5. Proposed COVID-19 Prediction Framework

**Figure 4:**
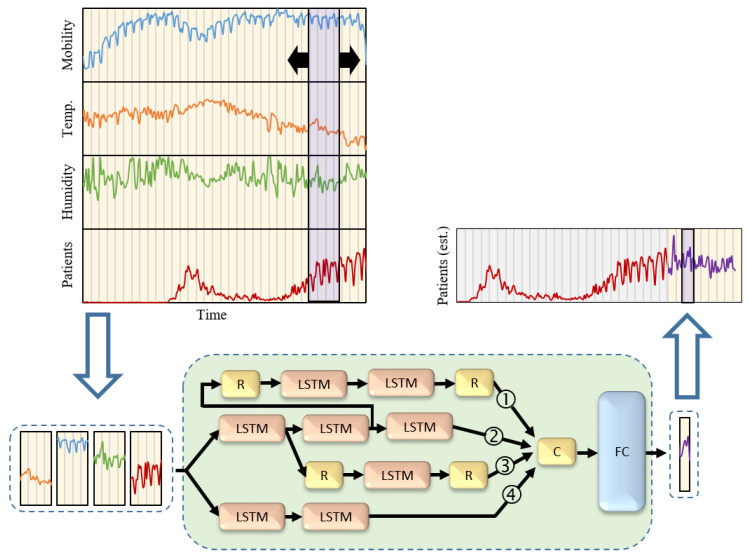
Proposed deep learning architecture. The LSTM network was trained using a set of time-sampled data consisting of mobility, maximum temperature, average humidity, and the corresponding recorded COVID-19 positive cases. R, C, and FC are sequence reverse, concatenation, and fully connected layers, respectively. Labels 1 to 4 demonstrate different paths used in the ablation study.

The principal idea of the proposed framework is the high correlation between the public mobility estimate and potential increase of SARS-CoV-2 infections. Crowded regions would lead to high probabilities of viral transmission [38,39]. Moreover, we believe that changes in meteorological factors have some useful insights that may improve forecasting results. The proposed framework is based on the LSTM neural network architecture [26]. A deep learning architecture with LSTM layers has proven to be an efficient design for time series data manipulation and regression. We consider a novel multi-path design in which data pass through different data processing pipelines for better feature extraction. The data extracted from all paths are then processed within an FC layer to estimate future positive cases. The training dataset was designed as a set of time-sampled values over *k* days with values of mobility, maximum temperature, average humidity and reported COVID-19 positive cases. These features were selected from our previous studies as the dominant factors correlated with COVID-19 positive cases. However, the extension to include other factors is simple and direct. The data mapping is formulated as follows:

(1) X→Y,

(2) $$X=(x_{1},x_{2},\cdots ,x_{k}),$$

(3) $$Y=(y_{k+1},y_{k+2},\cdots ,y_{k+l}),$$

(4) $$x_{t}=\left(m_{t},p_{t},h_{t},y_{t}\right),\;\;\;1\leq t\leq T,$$

where X and Y are the network input and output vectors, respectively, and $m_{t}$, $p_{t}$, $h_{t}$, and $y_{t}$ are the mobility, maximum temperature, average humidity, and number of recorded COVID-19 cases on day *t*. Obviously, a set of *k* days is mapped to the following *l* days for prediction, and *T* represents the number of days for all training datasets. This will enable the prediction of expected cases with accurate recorded data. A sketch diagram of the proposed network architecture and data flow is shown in Figure 4.

The network architecture was implemented with LSTM cells (each output vectors of size 300 elements) using Wolfram Mathematica (R) ver. 12.1 on a workstation with four Intel (R) Xeon CPUs running at 3.60 GHz, with 128 GB of memory and three NVIDIA GeForce 1080 GPUs. The FC layer was set as three linear layers of 600, 300, and 100 neurons each. The training was conducted using time frames of seven days (k=7, l=1) with the ADAM optimizer and cross-entropy loss function. For each data sample, all available data starting from 26 January 2020, up to one day earlier, were used for training for 2000 epochs and with a batch size of 32. The testing phase was repeated to estimate a set of future days as follows:

(5) $${\hat{y}}_{i+k}=Estimate\left(x_{i},x_{i+1},\cdots ,x_{i+k-1}\right),\;\;\;1\leq i\leq N$$

where *N* denotes the number of days in the test dataset. A single training experiment required approximately 5 min to complete.

### 3.6. Evaluation Metrics

For quantitative evaluation, the average relative error over a period of *N* days was computed as follows:

(6) $$E=\frac{1}{N}\sum_{i=1}^{N}\frac{|y_{i}-{\hat{y}}_{i}|}{y_{i}},$$

where $y_{i}$ and ${\hat{y}}_{i}$ are the real and estimated positive cases on day *i*, respectively.

## 4. Results

### 4.1. Prediction of Positive Cases

**Figure 5:**
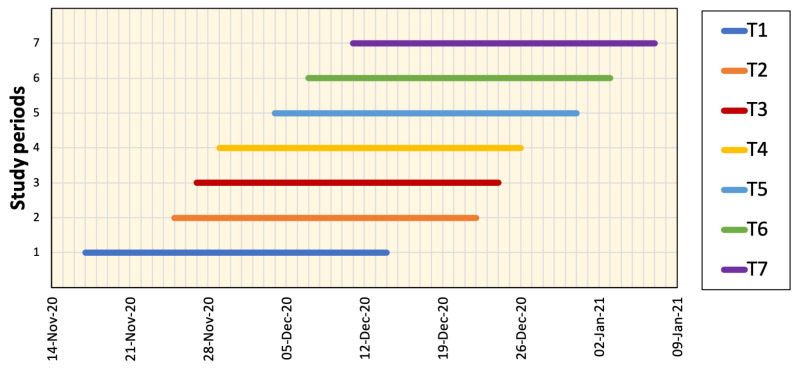
Different periods of prediction data (each of 28 days) investigated in this study.

To validate the proposed prediction method, we considered seven data prediction samples each of 28 days from 17 November 2020 to 7 January 2021, when the actual data were already available and the corresponding Google Cloud forecasting was announced (see Figure 5). The predictions released by Google Cloud regarding the daily number of positive cases in Tokyo, Aichi, and Osaka were compared to the proposed framework and actual numbers. Mobility reduction percentages were considered using NTT DoCoMo mobile data (from 1 May 2020 to 7 January 2021) in addition to Google mobility data (from 15 February to 30 April 2020) because NTT DoCoMo mobile data were not available for this time period.

**Figure 6:**
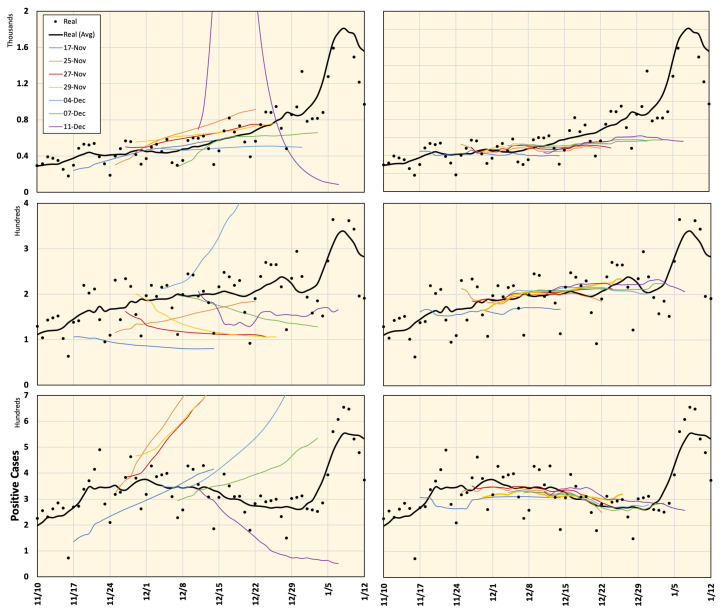
Prediction of COVID-19 positive cases in Tokyo, Aichi, and Osaka (from top to bottom) released by Google Cloud (**left**) and computed using the proposed framework (**right**), compared to actual real data for different time frames defined in Figure 5. All plots represent a 7-day average value.

**Figure 7:**
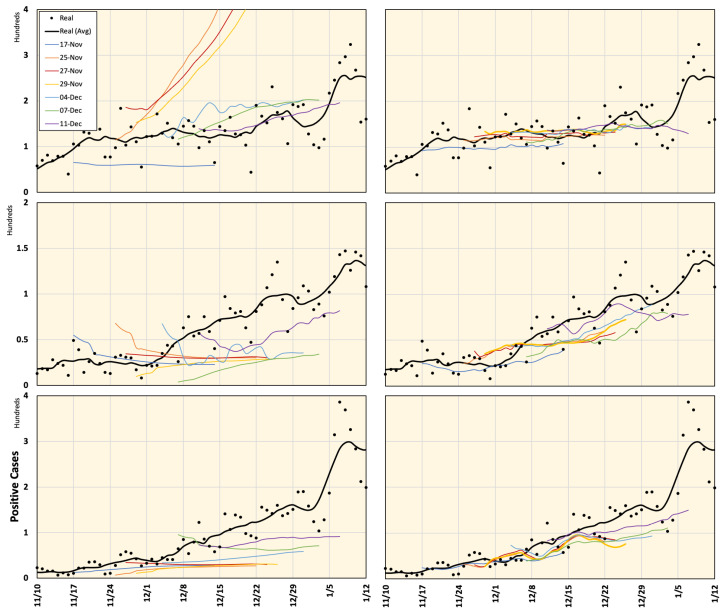
Prediction of COVID-19 positive cases in Hyogo, Kyoto, and Fukuoka (from top to bottom) released by Google Cloud (**left**) and computed using the proposed framework (**right**), compared to the actual real data for different time frames defined in Figure 5. All plots represent a 7-day average value.

**Figure 8:**
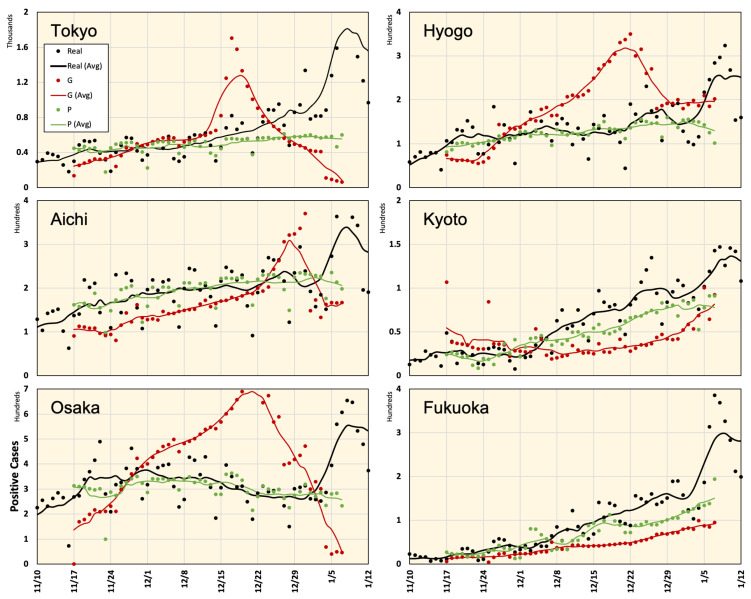
Real and estimated number of positive cases using Google Cloud forecast (G) and the proposed framework (P) for different regions in Japan. Values are computed as the average of the seven periods shown in Figure 6 and Figure 7, and the curves demonstrate a 7-day average.

The prefectures of Tokyo, Aichi, Osaka, Hyogo, Kyoto, and Fukuoka in Japan were considered in this study. The prediction results for these regions are shown in Figure 6 and Figure 7. As seen from these figures, Google Cloud forecasting with different time slots is highly inconsistent. However, the results of the proposed model were in good agreement with the actual values. Average estimated values from 16 November 2020 to 7 January 2021 using different methods are shown in Figure 8). One can observe that the forecasting data around the right-side peripheral in Figure 6 and Figure 7 are of low quality. With relatively long-term forecasting, error accumulation around the peripheral region is expected to be higher than that close to known data. In addition, this period demonstrated that end-of-year and new-year vacations occurred when special mobility activities occurred. In addition, the number of PCR tests reduced because most hospitals were closed from 28 December to 3 January suggesting that the number of new positive cases might have been reported in the week of 4 January 2021. Moreover, we believe that the proposed framework demonstrated a low-quality result during this period because there was no relevant history regarding this special season in the training dataset.

### 4.2. Influence of Meteorological Factors

**Figure 9:**
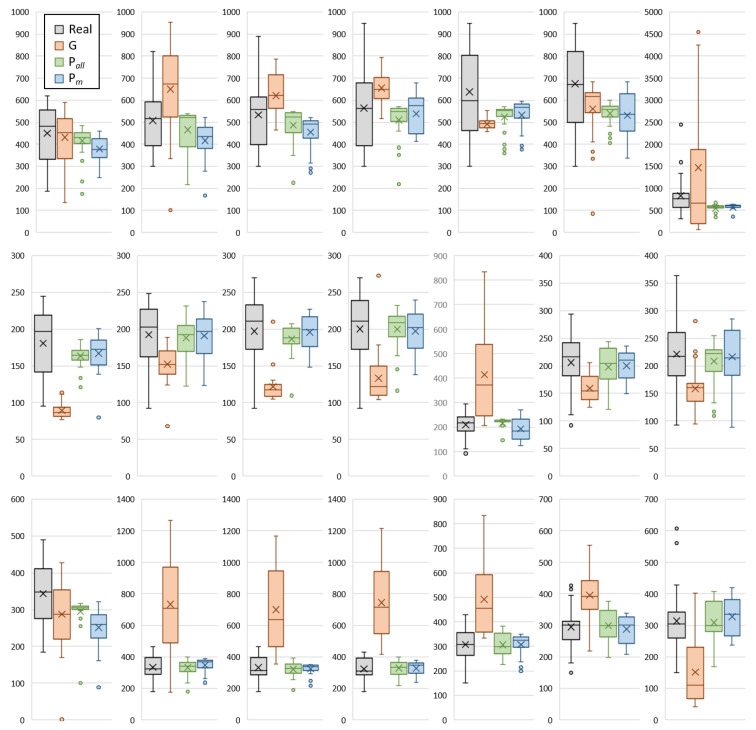
Boxplots demonstrate real (R) and estimated positive cases using Google Cloud forecast (G) and the proposed framework (mobility + meteorological factors) (P${}_{all}$) and mobility only (P${}_{m}$) for Tokyo (**top**), Aichi (**middle**), and Osaka (**bottom**). Plots represent seven time periods in chronological order from left to right.

To clearly demonstrate the efficiency of the proposed framework, we excluded meteorological data and repeated the experiment using only mobility data. The results are shown using the boxplot in Figure 9. This figure indicates that the results provided by the proposed framework with both sets of data (mobility only or mobility with meteorological factors) are superior to that provided by Google Cloud. For the numerical evaluation, the relative error values of the different methods are listed in Table 3. In Tokyo, the use of mobility data along provides the most accurate estimation, with an average relative error of 21.9%. In contrast, data from Aichi and Osaka demonstrate that mobility, temperature, and humidity achieve better estimations with average relative errors of 16.8% and 16.1%, respectively. In all cases, there was a significant improvement in the estimated values compared with the Google Cloud data.

### 4.3. Parameter Validation and Ablation Study

**Figure 10:**
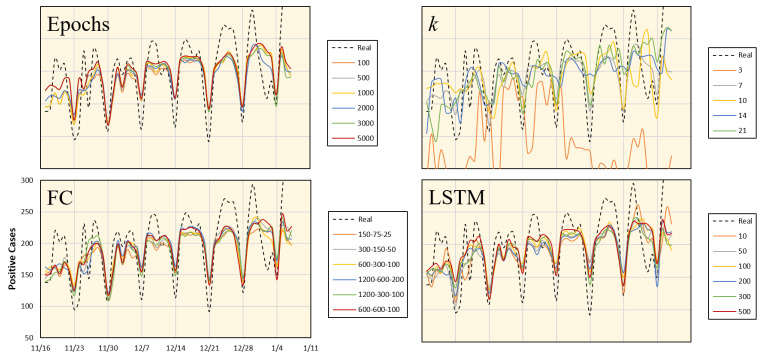
Number of positive cases estimated for the Aichi prefecture using different parameter settings.

**Figure 11:**
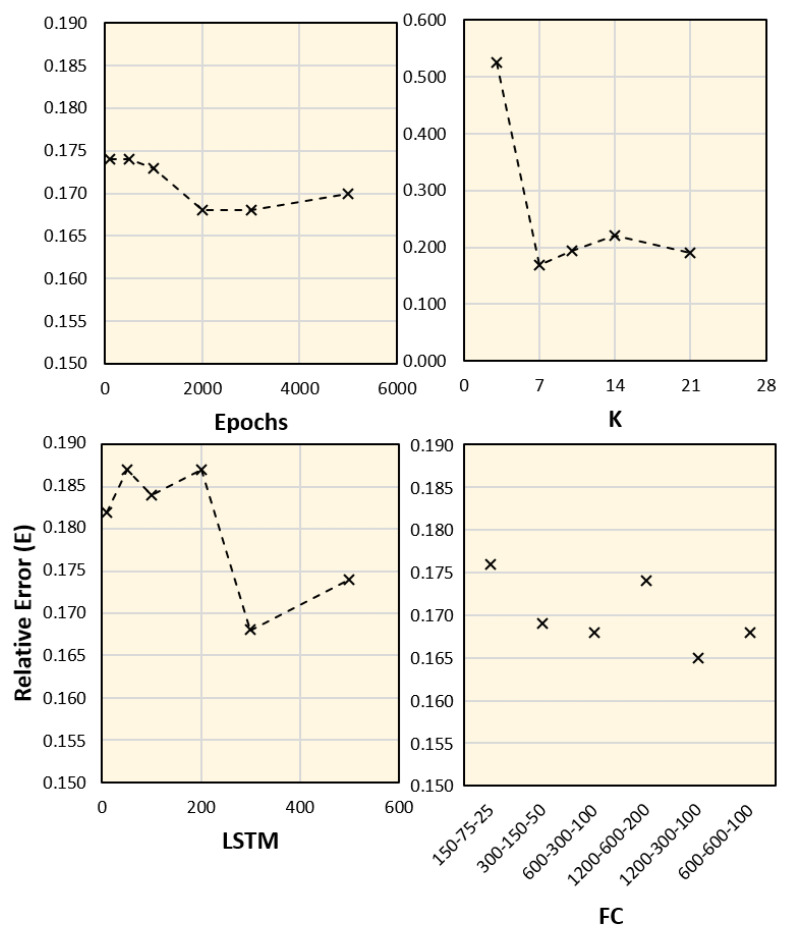
Relative error (*E*) for different parameter setups shown in Figure 10.

**Figure 12:**
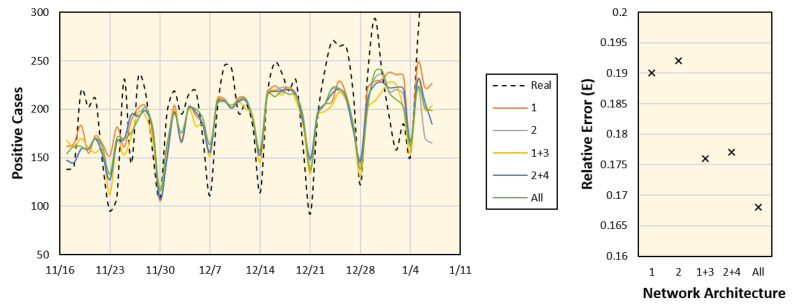
Number of positive cases estimated for Aichi (**left**) and corresponding errors (**right**) using different sub-networks as labeled in Figure 4.

An additional study was conducted to test the robustness of the proposed framework for different parameter setups, including variations in training epochs, *k*, and the sizes of the LSTM and FC layers. We considered data from Aichi, and the average values of the corresponding days were computed from the seven time periods. The results with different parameter setups and the corresponding error values are shown in Figure 10 and Figure 11, respectively. These results indicate that the most dominant parameter is the *k* value, which should be carefully adjusted. Moreover, an ablation study was conducted by customizing the network architecture. The number of network tracks (labeled from 1 to 4 in Figure 4) is reduced to only one or two tracks, as shown in Figure 12. It is clear from the figure that the full network performs better than the sub-networks; however, the error difference is marginal.

## 5. Discussion

With the uncertainty caused by the wide spread of the COVID-19 pandemic, there has been a high requirement for analyses that can provide insights on future situations. With high infection rates, healthcare services and facilities can be overburdened within a short period of time, which is known to have a negative social effect and increase fatality rates caused by COVID-19, as well as other chronic diseases that require special care. In 2020, many studies have presented different analyses of several factors and how they are related to the spread of COVID-19. However, future predictions regarding the spread of COVID-19 using long-term data are rarely discussed. An interesting step forward in this direction is the initiative by Google Cloud, which provides 28-day forecasting in the USA and very recently in Japan. Google Cloud data provide forecasting results that are matched with real data on many occasions; however, we found that it may be misleading as it provides data with high inconsistencies, specifically in the case of Japan. For example, it may be estimated that the number of cases will decrease, and after a few days, the forecast may indicate a curve in the opposite direction (see Figure 3).

With developments in modern communication technology, it has become easy to estimate public mobility, which can provide useful insights into urban areas [40]. The use of daily updated mobility data demonstrates a larger perspective of public reactions and is a good data source to understand potential risks based on public activities. With the help of machine learning techniques, processing such a blend of big data can lead to effective and timely decision making and public policy announcements [41]. Moreover, mobility data can be used to trace the public response to government policies, especially in the case of Japan, where a strict lockdown has not been enforced, and public response is voluntary.

Meteorological data such as temperature and humidity were also used as several earlier studies have shown that they are correlated with the spread of the pandemic. By merging meteorological factors with mobility estimates, we found that the proposed model can provide a slightly better estimation of COVID-19 positive cases. The results of Tokyo demonstrated a different trend, where the mobility data alone was sufficient for a more accurate estimation compared with those computed using mobility and meteorological factors (with a difference in the average error of 0.7%). This small value can be accepted considering the urban area structure of the capital with the highest population density and high commuting activities with neighboring prefectures. Moreover, there are several positive cases recorded in Tokyo owing to pandemic epicenters within hospitals and elderly nursing facilities [24], making proper comparisons difficult.

The correlation between the different factors discussed in this study is complicated. For example, an increase in public mobility is known to lead to more potential close distance contact that may lead to an increase in infection and vice versa. In contrast, an increase in infection usually leads to public alerts, which is likely to lead to a reduction in non-essential outdoor activities. A key factor in this correlation is the time lag and public response. Within this context, the proposed method can estimate the potential risk using feasible and accessible data. On 12 December 2020, a national Japanese newspaper (Chunichi Shimbun, ranked fourth in national distributions) used data generated using an earlier version of the proposed framework for public enlightenment, and it appeared as the top news on the first page (https://www.chunichi.co.jp/article/168959 (accessed on 13 December 2020)). The data presented in the newspaper are shown in Appendix A. The proposed framework is generalized so that it can be easily fit with different datasets that represent different factors associated with the spread of the pandemic.

## 6. Conclusions

In this study, we investigated a machine learning approach that includes mobility information as well as meteorological data within a neural network architecture that is trained to predict future COVID-19 positive cases. A set of seven time periods, each of 28 days, for six different prefectures in Japan were used for the assessment of the proposed framework. The proposed framework provided more accurate and consistent estimations than that provided by Google Cloud. Data represented positive cases in six prefectures in Japan can be predicted for different time frames with average relative errors of 22.6% (Tokyo), 17.1% (Aichi), 16.2% (Osaka), 26.3|% (Hyogo), 41.9% (Kyoto), and 38.9% (Fukuoka), which represent a range from 0.18 (best case) to 0.75 (worst case) of the average relative error of Google Cloud forecasting. Moreover, the forecasting patterns were almost consistent with the actual data in terms of the spread/decay phases. The datasets and/or software generated during the current study are available from the corresponding author on reasonable request.

## Figures and Tables

**Table 1:** Brief list of recent studies that demonstrate the use of LSTM network architectures in forecasting COVID-19 cases.

Ref.	LSTM Arch. ^1^			Data Range ^2^		Input Data	Region
	Sk	Bi	Cv	from	to		
[27]	✓			30 January	4 April	DPC	India
[28]	✓	✓	✓	14 March	14 May	DPC	India
[29]	✓	✓	✓	7 February	7 July	DPC	India/USA
[30]	✓			10 February	18 March	GT	Iran
[31]	✓			22 January	3 May	DPC & SDH	Isfahan (Iran)
[32]	✓			22 January	7 July	DPC	Russia, Peru & Iran
[33]		✓		22 January	31 March	DPC	Canada

^1^ Sk: stacked, Bi: bilinear and Cv: convolutional. ^2^ all dates are within 2020.

**Table 2:** Population, population density, total cases, and maximum DPC of study regions.

Region	Population (×1000)	Density (per km^2^)	Total Cases (till 7 January 2021)	Max. DPC (till 7 January 2021)
Tokyo	13,921	6354.8	69,140	2520
Aichi	7552	1460.0	18,332	431
Osaka	8809	4631.0	32,655	607
Hyogo	5466	650.4	11,193	284
Kyoto	2583	560.1	5518	143
Fukuoka	5104	1024.8	10,364	386

**Table 3:** Relative error (*E*) computed using different forecasting methods (Google Cloud (G), proposed with temperature, humidity, and mobility data (P${}_{all}$) and proposed with mobility data only (P${}_{m}$)) for all study regions over seven time periods ($T_{1}$–$T_{7}$) shown in Figure 5. Blue color indicates the lowest error value.

Region	Method	T_1_	T_2_	T_3_	T_4_	T_5_	T_6_	T_7_	Avg.
Tokyo	G	0.289	0.467	0.287	0.330	0.300	0.290	1.943	0.558
	P${}_{all}$	0.267	0.169	0.191	0.222	0.229	0.229	0.278	0.226
	P${}_{m}$	0.233	0.221	0.191	0.198	0.210	0.234	0.249	0.219
Aichi	G	0.467	0.301	0.368	0.395	1.102	0.311	0.376	0.474
	P${}_{all}$	0.201	0.148	0.189	0.162	0.150	0.157	0.187	0.171
	P${}_{m}$	0.184	0.149	0.172	0.174	0.149	0.174	0.232	0.176
Osaka	G	0.318	1.464	1.334	1.477	0.802	0.505	0.533	0.919
	P${}_{all}$	0.234	0.148	0.159	0.141	0.130	0.135	0.190	0.162
	P${}_{m}$	0.283	0.171	0.166	0.151	0.132	0.141	0.214	0.180
Hyogo	G	0.470	1.892	1.740	1.532	0.520	0.425	0.336	0.988
	P${}_{all}$	0.282	0.286	0.244	0.229	0.248	0.216	0.336	0.263
	P${}_{m}$	0.330	0.289	0.234	0.248	0.207	0.263	0.360	0.276
Kyoto	G	0.538	0.749	0.568	0.601	0.758	0.732	0.363	0.616
	P${}_{all}$	0.429	0.529	0.564	0.549	0.283	0.342	0.241	0.419
	P${}_{m}$	0.448	0.494	0.519	0.564	0.601	0.615	0.540	0.540
Fukuoka	G	0.484	0.665	0.506	0.663	0.535	0.443	0.353	0.522
	P${}_{all}$	0.399	0.420	0.461	0.428	0.429	0.341	0.245	0.389
	P${}_{m}$	0.519	0.427	0.388	0.358	0.335	0.334	0.375	0.391

**Figure A1:**
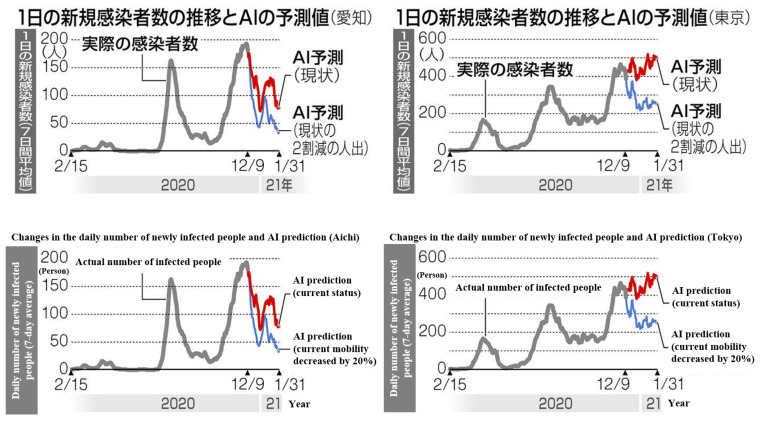
Estimated number of positive cases in Aichi prefecture (**left**) and Tokyo metropolitan (**right**). The red color indicates the case wherein there was no change in public mobility, and blue indicates the case where mobility reduced to values equivalent to August 2020. These plots were published on 12 December 2020. English version is shown below the original charts in Japanese.

## Data Availability

The datasets and/or software generated during the current study are available from the corresponding author on reasonable request.

## Appendix A

Data generated using an earlier version of the proposed framework were used for public announcements in the Japanese newspaper Chunichi Shimbun, and the results of Aichi and Tokyo are shown in Figure A1. The novelty of our proposal is the long-term prediction (two weeks or longer) considering different mobility and weather conditions. The average of the past five-year (2015–2019) weather data was used for predictions to make the public understand the amount of mobility reduction needed to suppress DPC.
